# Integrated database for economic complexity

**DOI:** 10.1038/s41597-022-01732-5

**Published:** 2022-10-15

**Authors:** Aurelio Patelli, Luciano Pietronero, Andrea Zaccaria

**Affiliations:** 1 Centro di Ricerca Enrico Fermi, Via Panisperna 89 A, I-00184 Rome, Italy; 2 Istituto dei Sistemi Complessi (ISC) - CNR, UoS Sapienza, P.le A. Moro, 2, I-00185 Rome, Italy; 3 Dipartimento di Fisica Università “Sapienza”, P.le A. Moro, 2, I-00185 Rome, Italy

**Keywords:** Complex networks, Developing world

## Abstract

We present an integrated database suitable for the investigation of the economic development of countries by using the Economic Fitness and Complexity framework. Firstly, we implement machine learning techniques to reconstruct the export flow of services and we combine them to the export flow of the physical goods, generating a complete view of the international market, denoted the Integrated database. Successively, we support the technical quality of the database by computing the main metrics of the Economic Fitness and Complexity framework: (i) we build a statistically validated network of economic activities, where preferred paths of development and clusters of High-Tech industries naturally emerge; (ii) we evaluate the Economic Fitness, an algorithmic assessment of the competitiveness of countries, removing the unexpected misbehaviour of economies under-represented by the sole consideration of the export of the physical goods.

## Abstract (summary)

**Table:** 

Measurement(s)	export flow of services and goods
Technology Type(s)	python routines

## Background & Summary

Economic Fitness and Complexity^1–6^ (EFC) is a conceptual and practical framework for the estimation of the competitiveness of nations and the relatedness between sectors, borrowing ideas and methods from Statistical Physics and Complex Systems Science^7^. A key feature of the method resides in its bottom-up, data-driven approach, which relies on methods based on complex networks and machine learning to reconstruct and investigate economic actors (e.g., countries, regions, firms) and their activities (e.g., export, patenting, scientific production). All the methodology is based on empirical observations and certifiable hypotheses. This approach departs from the canonical econometric narrative, where economic performances are usually gauged by monetary metrics, such as the Gross Domestic Production (GDP). Instead, EFC aims to capture the competitiveness of a country, and not its wealth, by introducing a synthetic and non-monetary metric, the Fitness^1^, bringing to light new and relevant economic patterns. We point out that the data hereby presented can be useful also for mainstream econometric investigations.

Economic Fitness and Complexity relies on homogeneous and high-quality data from which it effectively extracts information aiming to achieve a maximal signal-to-noise ratio. In the *Big-Data* era, the unprecedented large size of the new databases can generate broad confidence on the new possibilities offered by large-scale analytic, although issues related to the quality of the data itself are sometime underplayed^8^. Actually, it is rather intuitive that Big-Data can have “Big-Noise”^9^, and a careful selection of the sources must be accomplished at the starting point of the research in any data-driven field. The reference database in the classical EFC analysis is the International Trade database reconciled and regularized by Tacchella and collaborators^10^ starting from the UN-COMTRADE data. This database covers the external flows of physical Goods between reporting countries in the World (about 175 countries) with a very detailed classification, collecting more than 5000 distinct products. EFC is based on the export data for both conceptual and a practical reasons. On one hand, export data is rather homogeneous and standardized across different countries. On the other hand, the idea behind EFC is to derive the competitiveness of countries by inferring the presence of capabilities through an indirect way, i.e. by assuming that different sets of capabilities map to different productive baskets and then diversified exports are the signal for a competitive nation^11^. Not trivially, the GDP predictions obtained by EFC and based on the Trades indicate a quantitatively better performance compared to the best conventional economic models^10^ and with a much lower data requirement. This result represents an a-posteriori confirmation of the goodness of the EFC approach.

Unfortunately, the Services are not included in the set of available features in UN-COMTRADE, despite a relevant and growing fraction of money flows through the channel of the Services^12^, and the consequent importance for both developed and developing economies. A first attempt to complement the Services into the aforementioned analysis was followed by^13^, finding that at a very aggregated scale the Services tend to be more complex with respect to the Goods. However, the authors complain that the high level of aggregation may be the cause of a loss of the information necessary to accurately measure the Complexity of the Activities (i.e., Services and physical Goods) and the Fitness of the Countries. Using a finer aggregation scale, the authors in^14^ show a more heterogeneous scenario of the Complexity of Activities, pointing out that complex Services cluster with complex Goods. In the EFC narrative, clusters of Activities indicates that there is a participation of common intangible capabilities^3,15^. However, either manuscripts have to select a subset of the originally available economies, because the databases implemented in their analysis present many missing features and do not cover uniformly the nations. Indeed, both references consider about one hundred countries, with important missing economies such as China and Great Britain. Recently, the work of Mishra and collaborators^16^ shows the importance of a common database aggregating both Services and Goods, especially for a correct analysis of the economic relevance of the developing nations. Finally, Saltarelli *et al*.^17^ use the World Input-Output Database to show that export mirrors remarkably well domestic production for manufacturing sectors, but this relation fades away for Service related sectors.

The analysis endowed by the previous works highlight the purposes of accounting the Services in the correct estimation of the competitiveness of the nations. However, the reference database for the Services, the *Balance of Payments and International Investment* (BOP) data collected by the International Monetary Fund (IMF)^18^, presents some weakness and important missing scores, especially in comparison with the quality of the Goods database. Strikingly, the actual IMF-BOP classification is unsuitable for the EFC analysis, presenting an overlapping and convoluted hierarchy of sector. The first core result of the present paper is the reconciliation of the quality of two databases, the Goods and the Services, obtained by reclassifying the IMF services sectors and reconstructing the missing elements of BOP by using and comparing different machine learning techniques. The best reconstruction method obtained is then used to create the so-called *Integrated* database of EFC, aggregating Services and Goods for 160 countries and a total of 124 sectors, providing the largest set of common nations and sectors available from both the BOP and the 2digits UN-COMTRADE datasets. Finally, the Integrated database is used in the Economic Fitness and Complexity analysis, obtaining two conceptual and practical advances: i) a network of integrated sectors whose links are statistically validated, that will be of practical use to predict and recommend new sectors of development, and ii) the computation of the Universal Fitness, the novel EFC indicator to assess the competitiveness of nations, now including also the Services. We use the term Universal Fitness instead of Integrated Fitness in order to follow previous works^14^.

## Methods

This section introduces the Integrated database, aggregating Goods and Services; in particular, we describe the gathered data and we focus on methodologies we adopted for the re-classification of services and the reconstruction of the missing values. In the last section we discuss the data pre-processing methodology generally adopted in the EFC framework.

### Goods: the international trade of products

Following the definition of IMF^19^, a Good is a physical item or commodity over which ownership can be passed via transaction. Consistently, such transactions are recorded by the customs and available (on a monthly or yearly basis) via the UN-COMTRADE website^20^. Following the standard approach of EFC, the reference database reports the volume of export (in dollars) of each product exported by each country. This data can be organized in a matrix whose rows correspond to countries and columns to products.

The original data collects the export flows of classes of physical products between nations, this is then reconciled to obtain the export matrix described above. The temporal coverage of the Goods database runs from 1996 to 2018, spanning slightly more than two decades, and it is made available for 169 countries corresponding to the principal economic players in the World. The classification of the products we use follows the Harmonized System revised in 1992 (HS92), consisting of 5040 sectors labeled by 6-digits codes at the so-called sub-heading level. Note that the number of different products available at 6-digits is of order 5000, while the number of service sectors is around 30. The HS classification is updated every 5 years; remarkably, a relevant modification happens in 2007. Such modification induce a re-balance in some sectors at 6-digits that create a non-uniform transition in few low complexity codes.In order to overcome classification issues and obtain a more stable set of records, we aggregate the products from 6 to 2 digits by using the hierarchical structure of the classification. In this way we can also obtain a closest comparison to the Services database. Further, this choice balance the respective weights in the international trade^12^. The HS92 2-digits classification contains 97 codes, but we remove one sector (code 99, *Commodities not specified according to kind*) because it does not represent a defined activity or product and has a low impact on the overall exports.

### Services: description and classification issues

Accordingly to IMF^19^, a Service is the result of a production activity, or facilitates the exchange of Goods, or is a financial asset. Therefore, Services are usually non-separable items and cannot be detached from their production. The Service database we consider is based on the *Balance of Payments and International Investment* (BOP) data collected by the International Monetary Fund (IMF)^18^. BOP database offers three accounts for the Services-related transactions: the credit, the debit and the net values. In comparison with the measure available for the Goods, we select the credit indices, since they correspond to exported services. Further, two closely related classifications are present: the *full* BOP classification implemented by the IMF and an *alternative* alpha-numerical classification reported in their metadata. The full BOP classification is based on the composite nature of the codes of the Services but presents a key drawback for its usage in EFC: some sectors are not separated. In particular, from the hierarchical classification emerges the possibility that the sum of the sons of a code reports a total value of exports larger that the value assigned to the parent. This misleading situation happens because the classification may implement different methodologies in order to generate the hierarchy.

**Fig. 1:**
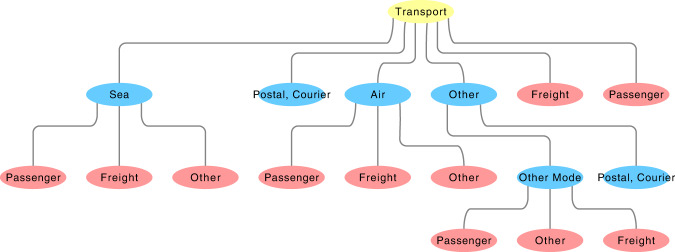
The network of the Transport Services and its sons divided with the two possible disaggregation of the codes. Only the blue codes sum up to the value of the parent. The name of the nodes corresponds to the last part of the definition available from the BOP’s metadata and the full definition corresponds to the sequential aggregation of the definitions of the parent nodes.

In order to explain this issue we discuss an example regarding the sons of the parent code ‘*Transport Services*’ (‘STR’ codes). In this case, there are two possible disaggregations: one considers the kind of transportation implemented, and the other the nature of the transported Goods. The full BOP classification implements both. In Fig. 1 we show the hierarchical network of the STR codes, highlighting the two disaggregation paths in blue and red. Remarkably, only the blue codes sum up to the value of their parents. Instead, the red nodes do not sum to the available value of the parents.

Contrarily, the alternative BOP classification maintains only a single hierarchical structure removing the double methodologies to generate the hierarchy. The internal consistency of this classification is corroborated by the fact that the sum of the values of the sons returns the value of the respective parent codes. We want to stress that the scenario of the two methodologies derives from a misleading interpretation of the indices offered by the IMF and not an error in their implementation of the database, since it is possible to reconstruct the correct structure by carefully investigating the metadata and manuals.

**Fig. 2:**
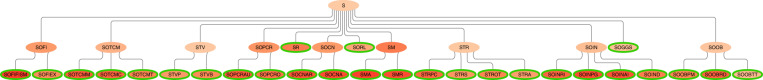
The tree graph of the relation between the BOP codes following the *alternative* classification. The colour of the nodes represents the frequency of missing elements found in the raw database with red corresponding to 1 and white corresponding to 0. The green circle indicates the codes considered in the final construction of the Integrated database (see text below).

In Fig. 2 we present the hierarchical structure of the alternative classification. Three layers, corresponding to as many levels of aggregation, are present. The first layer accounts for the total export from each country; its value is equal to the sum over all the sectors in the second layer and the sum over all the third layer. These two layers are more representative of the heterogeneity of services. In this work we will select the sectors circled in green; possible further sons of these sectors are not shown. Instead, our choice of the sectors tries to balance on one hand the possibility to consider the larger number of sectors increasing the level of details of the analysis and, on the other hand, the presence of reported values by the exporting countries. The latter obviously decrease as more detailed sectors are considered.

### Missing values in services data

The selection of the Service codes used in the Integrated database (Goods + Services) is shown in Table 1, and covers the lower levels of available aggregations, as highlighted in Fig. 2 by marking the codes with a thick green border. Hereafter, we will refer to this set of services as the *summable* code set, since knowing their values allow you to obtain values at coarser levels.

**Table 1:** The list of the 27 codes implemented in the summable set used in the Integrated database, with their description and original layer of aggregation.

code	layer	description
BXSOGGS	1	Government Goods and Services
BXSORL	1	Charges for the Intellectual Property
BXSR	1	Maintenance and Repair
BXSMA	2	Manufacturing Services, Goods for Processing Abroad
BXSMR	2	Manufacturing Services, Goods for Processing Inside
BXSOTCMT	2	Telecommunications Services
BXSOTCMM	2	Information Services
BXSOTCMC	2	Computer Services
BXSOPCRO	2	Cultural and Recreational
BXSOPCRAU	2	Audiovisual
BXSOOBTT	2	Technical, Trade-related, and Other Business
BXSOOBRD	2	Research and Development
BXSOOBPM	2	Consulting
BXSOINRI	2	Reinsurance
BXSOINPG	2	Pension
BXSOIND	2	Direct Insurance
BXSOINAI	2	Auxiliary Insurance Services
BXSOFIFISM	2	FISIM
BXSOFIEX	2	Explicitly Charged and Other Financial Services
BXSOCNA	2	Construction Abroad
BXSOCNAR	2	Construction Inside
BXSTVB	2	Travel Business
BXSTVP	2	Travel Personal
BXSTRS	2	Sea Transport
BXSTRPC	2	Postal and Courier
BXSTROT	2	Other Passenger Transport
BXSTRA	2	Air Transport

The BOP data represents approximately 200 countries (although, for this work, we select the subset of 160 countries that intersect the set of countries available for goods data), covering essentially all relevant economies of the world in terms of economic impact. Furthermore, in terms of temporal resolution, some BOP sectors date back to 1940, although initial data is rather sparse. However, we reconstruct the series starting in 1990 because the integrated database is constrained by the series of available Goods, which begins in 1996.

**Fig. 3:**
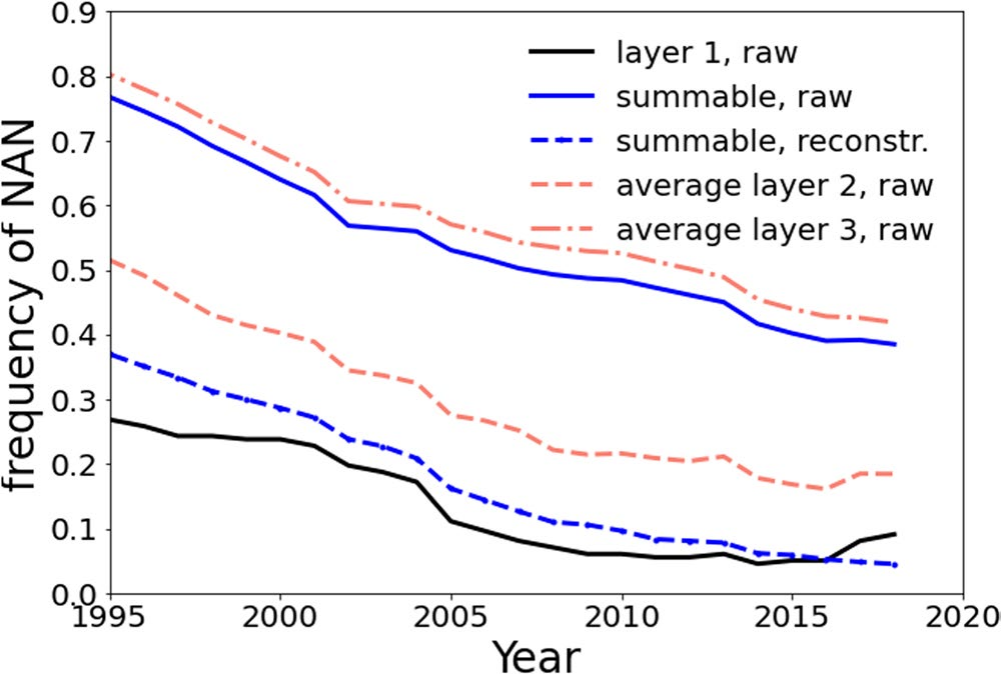
Proportion of missing elements (NAN) in the different possible sets of indices of the BOP database as a function of the years. The lines in red correspond to the different layers of the raw database while the blue lines correspond to the *summable* set in the raw (full line) and reconstructed using kNN (dashed line). The black line indicates the proportion of NAN in the upper layer 1.

Focusing on the temporal distribution of the missing elements, it is rather clear that the raw database is not uniform, as shown in Fig. 3, reporting the fraction of missing values (reported as NAN, not a number) as a function of time. Different lines correspond to different layers (i.e., levels of aggregation), classification (BOP or *summable*) and whether we used the kNN reconstruction technique (see below) or the raw data. Our reconstruction methodology allows for a drastic reduction of the missing values which makes our final data comparable with the quality of the upper layer.

In the early years of the series there is a larger presence of missing values with a decreasing global trend approaching the recent times. In the developed economies these behaviours are primarily induced by the complete absence of the first segments of the series for all the indices. For example, in the case for Austria, no information is available before 2005; regarding Belgium before 2004, and Japan before 2000. Instead, less developed countries present subsets of the indices with missing scores on short to medium temporal windows, while single missing values are rarer in the datasets. Hence, in between 2005 and 2015 the raw database offers its better quality, even if the raw version of the *summable* classification continues to have a high fraction of missing values.

Finally, also the geographical coverage of the raw database is not uniform, as shown in the top map of Fig. 5. Many countries have a large fraction of missing values, even if some of them are developed economies. Remarkably, Switzerland, China, Great Britain, and Spain are among those developed countries with a low quality representation in the raw data. This situation of heavy lack of data and heterogeneity calls for a specific intervention to reconstruct the missing elements, which is the subject of the next section.

### Reconstruction of the missing Services

In this section we discuss the reconstruction of the services database using different machine learning techniques, whose final output is then assessed and compared. In principle, the use of the alternative classification discussed above would allow the derivation of the full hierarchy and, as a consequence, the reconstruction of the *summable* subset of services by summing the respective indices (green-circled nodes in Fig. 2) if all sectors of the last layer were known. This is actually not the case, so we proceed in the opposite direction, by using the values of the upper layers to reconstruct the lower. This also allows for a higher stability of the relative importance of the nations or of the service sectors. Moreover, we assume that any not-missing value of the raw database is *correct*; in particular, also the entries with value equal to zero are labelled as correct, while negative entries are set to zero, although they are very few (are the 0.06% of the total number of entries).

A very basic and easily interpretable reconstruction technique is given by the linear interpolation of the temporal series in the forward direction, *i.e*. generating future elements for which there exists some previous information in the past. The linear interpolation is assumed as the reference method, giving relatively good results despite the simplicity of the method. Successively, we consider other two methodologies: a suitable machine learning algorithm, Temporal Random Forest^21^ and the Nearest-Neighbour imputations^22^.

The Random Forest (RF)^23^ is one of the most popular machine learning algorithms for classification and regression. The training leverages a set of samples for which both features and answers are known (here, the export volumes). Once the model is trained, it can be used to predict, given a set of features, a new answer whose value is unknown (in our case, an export volume to reconstruct). RF consists in an ensemble of decision trees^23^, single and non-overlapping subdivisions of the space of the features, *i.e*. the values of the series, into distinct regions following the hierarchical ordering provided by the progressive feature-selection, which results in a tree structure^24^. Since the specific features of the single trees are randomly chosen, it is possible to derive many different decision trees, which constitute a statistical ensemble and prevent overfitting. Using the RF it is possible to estimate each missing element of the input database (here the Services) based on the behaviour of the other entries for which those features are specified. This method is applied by taking as features the known export volumes both at different times and spaces (i.e., years and countries). This approach is called Temporal Random Forest^21^; the idea is to leverage the information from similar economies and years to predict the missing values. In ref. ^21^ this method is applied to a different database, and it is found to provide lower reconstruction errors with respect to other methods, and in particular the separate reconstruction of each country time series.

The latter method we implement is the k-Nearest-Neighbour (kNN) imputation^25^. It consists in the generation of the missing values from a weighted network average from the known values. The first step is the construction of a network of countries for each year. The weight of the link between each couple of countries is defined as their Euclidean distance; the countries here being defined by the export volumes relative to the upper known layer of services sectors. In this sense, two neighbour countries will share similar exports in the known sectors. Thus, the assumptions is that the reconstruction does not strongly modify the relative distance of the countries. Each missing value refers to a country c - sector s pair. The similarity network is applied for the selection of the countries that are closer to c and report an export volume for s. The kNN imputer^25^ reconstructs the missing value as the weighted average of the declarations of the same sector s given by the first *K* nearest-neighbours countries. The weights are given by the inverse of the Euclidean distance. Finally, we implement a check of the hierarchical coherence, consistently harmonizing the flows of child and parent codes on all the reconstruction techniques.

**Fig. 4:**
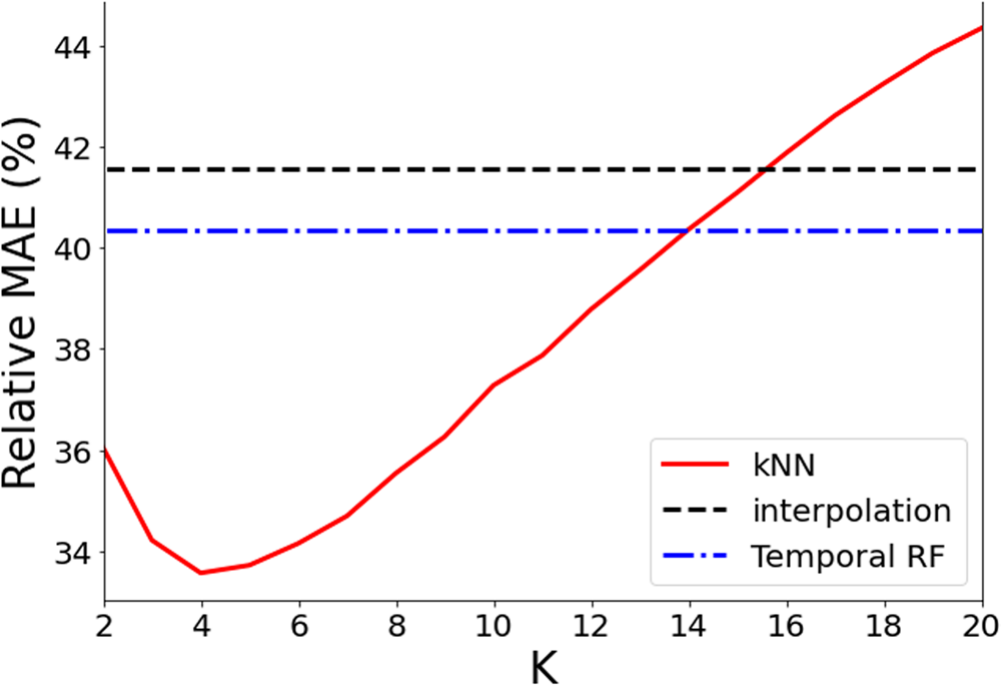
The relative Mean Absolute Error of the proposed reconstruction techniques as function of the number of Nearest-neighbours *K*, with respect to the total amount of economic flows.

We test and compare the quality of the different reconstructions on a subset of the database composed by countries and sectors for which all entries are specified. From this dataset we remove 100 elements randomly chosen, and we repeat this procedure 100 times, obtaining 100 samples with random missing values to reconstruct. The quality of the reconstructions are finally evaluated by the computation of the relative Mean Absolute Error (MAE) between the reconstructed and the ground-truth values. The relative MAE is evaluated with respect to the total amount of economic flow and, as shown in Fig. 4, the Temporal Random Forest outperforms the interpolation method, and the best reconstruction is obtained by using a kNN imputer which considers K~4–5 neighbours. Remarkably, the MAE is very large and it is mainly due to the fact that the raw data has a low quality.

**Fig. 5:**
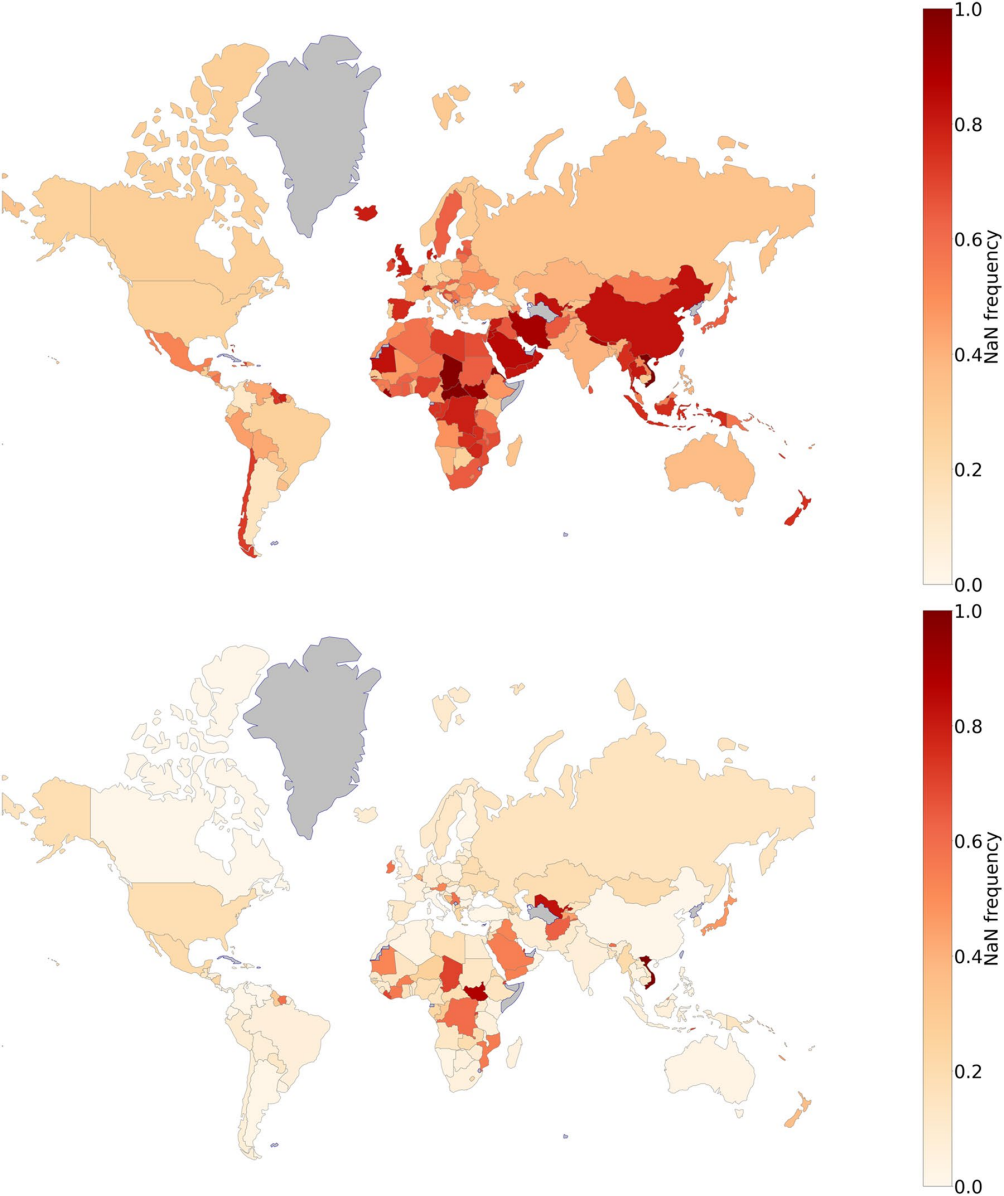
Maps of the World where the colour corresponds to the density of missing elements in the *summable* set of indices of the database of the Services. The top map shows the density in the raw data while the bottom map shows the density in the reconstructed data.

Therefore, in order to reconstruct the database we use in the Integrated database, we implement the kNN method with *K* = 5 neighbours to reconstruct the *summable* set of services described in the previous section (we may expect that increasing the size of the numerical test requires a larger *K* because it would be more probable to find closer and better neighbors, but the difference in MAE between 4 and 5 is not appreciable). Figure 5 shows World maps drawing the colours of the countries in terms of the fraction of missing elements in the raw (upper figure) and reconstructed (lower figure) databases and allowing a visual representation of the amount and quality of the reconstruction.

In order to obtain the Integrated database, we consider the reconstructed database of services only between the years 1996 and 2018 and we aggregate it to the 2-digits UN-COMTRADE data.

### Revealed comparative advantage and market share

The various Economic Complexity measures are not directly computed from the export volume but starting from quantities derived from it but normalizing the output. In this section, we discuss this normalization issue and derive the Revealed Comparative Advantage (RCA) and Market Share (MS) matrices which, in turn, will be the starting point to compute the EFC metrics.

The raw export volumes are not directly used in the EFC analysis for two reasons: i) they trivially depend on both the sizes of the sector and the country and ii) they do not provide a uniform assessment of the *competitiveness* of the given country in exporting a product. In order to overcome these limitations, the Revealed Comparative Advantage (RCA), introduced by Balassa^26^, is commonly used in the literature^1,27^. In formula, the RCA of country *c* in activity *a* is computed as:

1 $${{\rm{RCA}}}_{c,a}=\frac{{E}_{c,a}/{\sum }_{a}{E}_{c,a}}{{\sum }_{c}{E}_{c,a}/{\sum }_{c,a}{E}_{c,a}}$$

where *E*_*c,a*_ is the value of the Product or Service sector *a* (what we generically call an activity), in constant US dollars, exported by country *c*. Note that this formulation permits to identify a natural threshold of 1 to determine whether *c* exports *a* in a competitive way or not with respect to both the total export of *c* and the total market of *a*. We can then define the binary matrix *M* whose element *M*_*c,a*_ is equal to 1 if RCA_*c,a*_ ≥ 1, and it is equal to 0 otherwise.

We also define the Marked Share (MS) matrix element as:

2 $${{\rm{MS}}}_{c,a}={E}_{c,a}/\sum _{c}{E}_{c,a}$$

that is an assessment of the importance of country *c* in the global trade of *a*. Note that in this case no natural threshold is available, and a residual correlation with the size of the country (namely, its population, or total GDP) is usually present. Hence, we call the Fitness based on *MS* the Extensive Fitness, recalling the concept of extensive and intensive quantities in statistical physics, and we indicate the *standard* computations of the Fitness based on the binary RCA as intensive for clarity.

Both the series of the Market Share and of the binary RCA present their sources of noise and different techniques can be implemented in order to reduce their negative effect^10^. However, for the sake of simplicity and since the data at the aggregated level of 2-digits usually consider large volumes, in which a large part of the fluctuations have already been summed out, in both the series of MS and RCA we apply a simple exponential smoothing with half-life of 3 years. The use of the exponential smoothing allows the reduction of the noise and has the positive side effect of maintaining a persistence of a few years in the temporal series, without the use of more complex Machine Learning models.

## Data Records

In this section we explain the data record associated with this work, including the repository where this information is stored, and we provide an overview of the data files and their formats.

The database is composed by the aggregation of the export of Goods and Services, the latter being reconstructed using the methods described in the previous section. The single parts of the databases are placed in the relative folders: the export data is in the *export* folder, the raw and reconstructed BOP is in the *service* folder and the aggregation with the binary matrices is located in the *universal* folder. We complement the data with the results of the EFC metrics: the Fitness and Complexity for both the intensive and the extensive measures in the *fitness complexity* folder and the statistically validated network of sectors in the *network* folder. For the sake of completeness, we also include in the repository the Economic Complexity Index^27^ metrics computed using the ecomplexity python library provided by Harvard University, although we do not discuss the relationship between ECI and Fitness in the main text because it is out of the scope of the present work.

All the databases and the files containing the results of the Fitness and Complexity algorithm are available in the *csv* (comma-separated-values) format of Unicode (UTF-8) string format. The first column contains the row indexes while the first row contains the column indexes. This choice of the format allows the implementation of further analyses using various open-source libraries and platforms.

The statistically validated network of sectors are proposed in the common GraphML format (http://graphml.graphdrawing.org/).

The datasets generated and analysed during the current study are available in the *Integrated database* repository^28^, 10.6084/m9.figshare.20167700.v1. Also, a public repository managed by Enrico Fermi Research Center hosts the present and future updates of the Integrated database, available at the url https://efcdata.cref.it/.

## Technical Validation

In this section we support the technical quality of the database by computing the standard EFC metrics, namely a statistically validated network of Relatedness and the Economic Fitness of countries.

### Relatedness assessment: network of sectors

Firstly, we assess the *Relatedness* between economic sectors, a measure of the overlap among the capabilities needed to produce them^29^. Usually, the output is a network of products, such as the Product Space^27^ or the Taxonomy Network^30^. In this paper, we will compute the Product Progression Network^14^, based on the Assist Matrix - BiCM validation framework introduced in^31^, because of two main advantages: (i) the time evolution is explicitly taken into account, permitting the construction of a *directed* network of sectors, and (ii) each link is statistically validated by comparing its weight against a distribution arising from a suitable null model. A measure of the relatedness between two activities can be obtained considering their co-occurrences, that is, by counting how many times each pair of activities is present in the baskets of each nation. To avoid the dimensional effect induced by the nested nature of the system, that is, the fact that developed countries are competitive in many activities and some activities are more widespread than others, we appropriately normalize simple co-occurrences with respect to both degrees. Following^31,32^, this is equivalent to calculating the probability that information flows from one activity to another as a random walker. The resulting weighted, monopartite network, obtained projecting the original bipartite structure into the activity layer, is called the Assist Matrix

3 $${B}_{a,a{\prime} }(t,\Delta )=\sum _{c}\frac{{M}_{ca}(t)}{{u}_{a}(t)}\frac{{M}_{ca{\prime} }(t+\Delta )}{{d}_{c}(t+\Delta )}.$$

where ${u}_{a}=\sum _{c}{M}_{ca}$ indicates the ubiquity of the economic sector *a* and ${d}_{c}=\sum _{a}{M}_{ca}$ indicates the diversification of the country *c*. Note that the temporal evolution of **M** is explicitly taken into account, so in principle one has a different network of activities for each *t* and Δ. Moreover, this network is almost fully connected, and the presence of spurious links prevents also simple tasks such as a clear visualization of the connectivity. In order to filter the links maintaining only the relevant ones, we have to statistically validate each link against a null model. The implementation of the statistical validation of the network is pursued by considering an ensemble of random networks preserving few suitable macroscopic constraints, setting constant average degrees of the nodes^33^. In detail, in the present work the random ensemble considered for the validation of the network of interaction is the Bipartite Configuration Model^34,35^ (BiCM). The BiCM randomizes all the information available but the degrees of the nodes, *i.e*. the ubiquity and the diversification, which are kept constant (on average). Indeed, activities with a larger ubiquity have higher probability to be produced by a random country by chance and the BiCM contemplates this information. So we generate the ensemble of randomized bipartite networks constrained to have the same degree sequences (on average) of the empirical network. These networks are used in turn to generate an ensemble of random Assist Matrices (at fixed (*t*, Δ)). The filtering procedure of the empirical network is performed as follows. We consider for each link the distribution of the weights of the same link in the random matrices. We seize only those links in the empirical Assist Matrix that are above a percentile threshold of 95%; these links define the statistically validated matrix at fixed (*t*, Δ). Successively, we further select only those links that are statistically significant on all the available years *t* at a fixed Δ, aiming to reduce the false inference problem. Therefore, for each Δ we have one topological graph keeping only the links that are found relevant on all the temporal series analyzed. The collected networks span 10 years of possible time delays (Δ∈[0, 10]). We consider the presence of auto-correlation on the temporal series of the raw signal, and therefore we collapse these graphs into a single network where each link has a weight equal to the number of times it has been validated at different Δ. This choice partially accounts for the multiple hypothesis issue, since links with a large weight are validated more often, and have a lower probability to be a false positive.

**Fig. 6:**
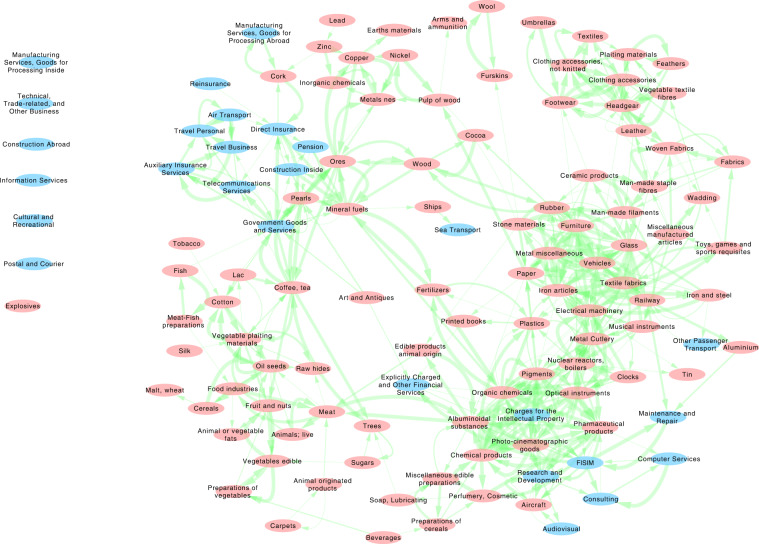
The statistically validated network of sectors. Goods are in pink, while Services are in light blue. The width of the links is proportional to the number of statistically validated time intervals.

Figure 6 shows the resulting network. On the bottom right, a High-Tech cluster contains both high Complexity Goods and Services. Going counter-clockwise, immediately above we find all the Heavy Industries and then Textile related sectors. Few other clusters collects the mineral Raw Materials and the Vegetable and the living Materials. These sectors show a lower degree of connectivity, indicating that countries specialized in these sectors rarely jump to more complex industries. The Services are not strongly dispersed but are not forming a single block of nodes. Note that High-Tech related Services, those associated to royalties and R&D, are strongly connected with the high Complexity sectors. Regarding only the Services, solely the group of transport nodes create a cluster *per se*. Finally, the statistical nature of the construction of the network allows that few nodes are not connected to any elements because their links are not found to be statistically relevant. A lower percentile threshold may connect them at the price that the final network will be less statistically significant with more likely erroneous inferences.

### Economic validation of the database: fitness and complexity

In this section, we use the Integrated database to compute the Fitness of countries and the Complexity of the Economic sectors. Different metrics to assess the economic complexity of countries exist;^1,27,36^ all of them can be computed from our database. For a broad discussion on the differences in both the spirit and the practical results we direct the reader to the relevant literature^37–40^; herein we only emphasize the predictive power of the Fitness approach. As shown in^10^, Fitness-based methodologies outperforms the state of the art (IMF) in forecasting the GDP time evolution.

The Fitness and Complexity algorithm^1,3,41^ aims at assessing the competitiveness, or *Fitness*, of a country, and the sophistication or *Complexity* of products using a set of coupled equations. The idea is the following: each country is characterized by its endowments, or capabilities, which represent its social, cultural, and technological structure^42^. These capabilities are expressed in what a country produces and exports, so the number of active sectors (physical Goods and Services) and their Complexities are linked to the Fitness of each country; in particular, the Complexity of a sector increases with the number and the quality of the capabilities needed in order to be competitive in it, and the Fitness is a measure of the Complexity and the number of the competitively exported sectors. In order to make this line of reasoning more quantitative, we start by considering the global structure of the matrix *M* defined above. Once countries and products are suitably arranged, the matrix M is triangular, or nested^43^, showing that developed countries have diversified exports, while less developed countries export fewer, lower Complexity products, and these products are actually the ones exported by all countries. In order to leverage on this structure to extract information about countries’ competitiveness and sectors’ Complexity, Tacchella *et al*.^1^ proposed the following set of non-linear, coupled equations

4 $$\left\{\begin{array}{lll}{\widetilde{F}}_{c}^{(n)} & = & \sum _{a}{M}_{ca}{Q}_{a}^{(n-1)}\\ {\widetilde{Q}}_{a}^{(n)} & = & \frac{1}{{\sum }_{c}{M}_{ca}\frac{1}{{F}_{c}^{(n)}}}\end{array}\right.\left\{\begin{array}{lll}{F}_{c}^{(n)} & = & \frac{{\widetilde{F}}_{c}^{(n)}}{ < {\widetilde{F}}_{c}^{(n)}{ > }_{c}}\\ {Q}_{a}^{(n)} & = & \frac{{\widetilde{Q}}_{a}^{(n)}}{ < {\widetilde{Q}}_{a}^{(n)}{ > }_{a}}\end{array}\right..$$

Here <·>_*x*_ denotes the arithmetic mean with respect to the possible values assumed by the variable dependent on *x*, with initial condition:

5 $${Q}_{a}^{(0)}=1\,{\rm{\forall }}a.$$

The iteration of the coupled equations leads to a single fixed point that does not depend on the initial conditions^1,3,41^. The fixed point defines the non-monetary metrics quantifying the Fitness *F*_*c*_ and the Complexity *Q*_*a*_. The convergence properties of Eq. (4) are not trivial and have been extensively studied by Pugliese *et al*.^41^. The coupled equations in (4) relies only on one input, the matrix *M* stating which countries are competitive in which sectors. Usually, the matrix is represented by the binary RCA, although also the Market Shares can be entered in Eq. (4), return the Extensive Fitness^1,14^. The immediate consequence of this choice is a higher correlation with the size of the country, as expressed for instance by its GDP.

**Fig. 7:**
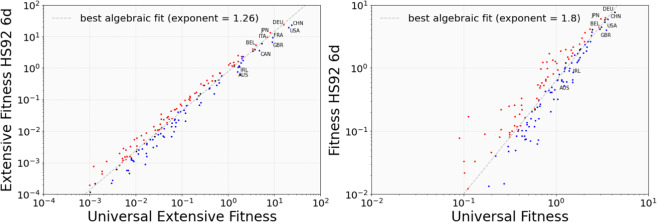
6d vs 2d and with/without services comparison. On the left, the effect on the Extensive Fitness values of integrating services and aggregating into 2 digit sectors. On the right, the same analysis performed using the intensive Fitness. In both cases a good correlation is present, and the major deviations are given by services or goods-driven Economies. The dashed lines indicate the least square regression fit with a power law shape, while the colour scheme highlights the countries gaining ranking positions in red and losing position in blue.

**Fig. 8:**
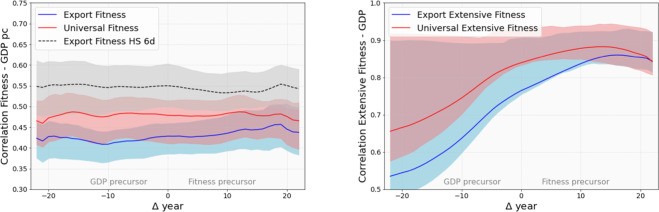
Extensive fitness as GDP precursor. Average correlations (Pearson coefficient) among the GDP and Fitness series with temporal lag. The left panel has the intensive measures while the right panel has the extensive ones. The filled regions is the region in between the 25% and the 75% quantile from the application of the bootstrap.

In Fig. 7 we compare the results of the Fitness algorithm when different input matrices are used. On the left we compare the Universal (i.e., considering Goods and Services) Extensive (i.e., the Market Shares matrix *MS* is used) Fitness, on the x-axis, with the Extensive Fitness computed at 6-digits, that is, considering only the Goods and the lower available level of aggregation, containing about 4500 different codes. On the right, instead, we show the intensive counterpart, that uses as input the binary RCA. By visually inspecting the figure one can notice various properties. First, a rather good correlation is present, highlighting that the overall economical results based on the Fitness metrics are robust when Services are integrated and the sectors are aggregated from the 6 to 2 digits levels. More importantly, the positive effect of specializing in high Complexity Services clearly emerges. Countries such as Great Britain (GBR) and Germany (DEU) provide two examples of Services or manufacturing driven countries, as correctly reflected by the universal Fitness indicator. As shown in refs. ^10,44^ the Fitness metrics can be used to forecast the GDP variations with a relatively long time interval and high accuracy. Hence, in this work we address a basic analysis in which we consider the time delayed cross-correlation between the Fitness measures and the respective GDP values with the aim to evaluate if the aggregation of the Services possibly introduces more signal in the forecasting. In Fig. 8, on the left, we plot the correlation between the intensive Fitness and the GDP per capita (GDP pc), and the error bars are obtained by bootstrapping the countries. One can easily see that the lines are essentially flat, indicating that, albeit correlated, there is no clear time direction from Fitness to GDP pc or vice-versa for either measure. Interestingly, this is the case of also the 6-digits Fitness usually implemented in the GDP analysis. The highest correlation with GDP pc is achieved by 6d Export Fitness, although comparing the two incides at the same level of aggregation, here the 2-digits on exports, it is clear that the presence of services account a better correlation with the standard economic performances. On the right, we show the extensive counterpart: the Extensive Fitness, i.e. computed using the Market Shares, and the total GDP, not normalized using the population. The Extensive Fitness, and in particular the Universal Extensive Fitness, that contains both physical Goods and Services, predicts the GDP with a time delay of up to 20 years, that is of the order of the maximal extension of our data. Note that we are using the term prediction in the Economic sense, *i.e*. this not an out of sample forecast but a time delayed correlation. In this sense, we can safely say that the Universal Extensive Fitness is able to statistically anticipate, or is a precursor of, the total GDP. However, the opposite is not entirely true: despite there being a correlation signal, GDP has much lower predictive power and Fitness is somewhat more difficult to anticipate.

**Fig. 9:**
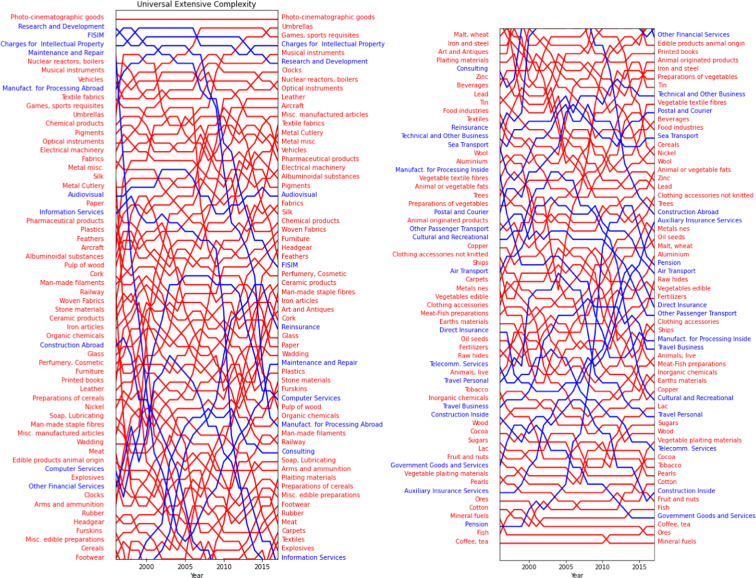
The series of the complexity ranking of the Integrated Sectors. The red colors refer to the physical Goods while the blue colors indicates the Services.

Finally, we show in Fig. 9 the time evolution of the Complexity rankings for both Goods (in red) and Services (in blue). Even if some noise is present, the rankings clearly follows the layman’s intuition of which sector may be sophisticated and which is not. In the high Complexity rankings we find services such as R&D and royalties, and manufacturing sectors such as nuclear reactors, optical instruments, and aircraft. Instead, low Complexity products correctly correspond to lower capabilities requirements such as Agrifood sectors. Note that some top code such as ‘Games, sport requisites’ includes also accessories and sophisticated products necessary for any kind of sport and competition, while others codes like Umbrellas are boosted by the fact that more than 83% of their Trade is performed by China.

## Usage Notes

The repository contains the Jupyter notebooks written in Python 3 necessary to reproduce the reconstruction and the Fitness and Complexity algorithm. Each notebook consist in a series of simple task:

- **raw aggregation.ipynb** notebook about the construction of the raw database into the format useful for the others computations (This requires the database downloaded from the IMF)
- **reconstruction service.ipynb** notebook about the reconstruction of the Services with different techniques
- **aggregate universal.ipynb** notebook about the aggregation of Goods and Services into the Universal database
- **int network.ipynb** notebook about the construction of the interaction network of the Universal features
- **fitness complexity.ipynb** notebook about the Economic Fitness and Complexity analysis

## Data Availability

The repository https://efcdata.cref.it/ contains the Jupyter notebooks written in Python 3 necessary to reproduce the reconstruction and the Fitness and Complexity algorithm.
